# Identifying optimal candidates for local treatment of the primary tumor among patients with de novo metastatic nasopharyngeal carcinoma: a retrospective cohort study based on Epstein–Barr virus DNA level and tumor response to palliative chemotherapy

**DOI:** 10.1186/s12885-019-5281-5

**Published:** 2019-01-21

**Authors:** Xue-Song Sun, Li-Ting Liu, Sai-Lan Liu, Shan-Shan Guo, Yue-Feng Wen, Hao-Jun Xie, Qing-Nan Tang, Yu-Jing Liang, Xiao-Yun Li, Jin-Jie Yan, Jun Ma, Qiu-Yan Chen, Lin-Quan Tang, Hai-Qiang Mai

**Affiliations:** 1 Sun Yat-sen University Cancer Center, State Key Laboratory of Oncology in South China; Collaborative Innovation Center for Cancer Medicine, Guangdong Key Laboratory of Nasopharyngeal Carcinoma Diagnosis and Therapy, Guangzhou, 510060 China; 2 Department of Nasopharyngeal Carcinoma, Sun Yat-sen University Cancer Center, 651 Dongfeng Road East, Guangzhou, 510060 People’s Republic of China; 3 Department of Radiation Oncology, Sun Yat-sen University Cancer Center, Guangzhou, 510060 People’s Republic of China

**Keywords:** Metastatic nasopharyngeal carcinoma, Epstein–Barr virus DNA, Tumor response, Local treatment, Radiotherapy, Survival

## Abstract

**Background:**

To evaluate the clinical outcome in patients with de novo metastatic nasopharyngeal carcinoma (NPC) treated or not treated with locoregional radiotherapy (LRRT) based on plasma Epstein–Barr virus (EBV) DNA level and tumor response after palliative chemotherapy (PCT).

**Methods:**

From 2007 to 2016, 502 patients with de novo metastatic NPC were included in this study. All patients were treated with PCT and 315 patients received LRRT. Our primary study endpoint was overall survival (OS).

**Results:**

EBV DNA was detected in 461 patients (91.8%) before treatment but was undetectable in 249 patients (49.6%) after PCT. Three hundred and seventeen patients (63.1%) achieved satisfactory response (complete response or partial response) to PCT. Both the post-PCT EBV DNA level and tumor response were independent prognostic factors. Among low-risk patients (patients with undetectable EBV DNA and satisfactory tumor response after PCT), the 3-year OS rate was 80.4% in LRRT-treated patients and 45.3% in patients not treated with LRRT (*P* < 0.001). Multivariate analyses demonstrated that LRRT was an independent prognostic factor of OS in the low-risk patients (*P* < 0.001). However, among the high-risk patients (patients with detectable EBV DNA and/or unsatisfactory response after PCT), no statistically significant survival differences were observed between the LRRT and non-LRRT groups.

**Conclusions:**

EBV DNA level and tumor response after PCT both correlate with the prognosis of de novo metastatic NPC. In such cases, LRRT may benefit the patients with undetectable EBV DNA levels and satisfactory tumor response after PCT.

**Electronic supplementary material:**

The online version of this article (10.1186/s12885-019-5281-5) contains supplementary material, which is available to authorized users.

## Background

Nasopharyngeal carcinoma (NPC) is a malignancy endemic in Southern China, where about 50–80 cases per 100,000 populations are reported each year [1]. According to its geographic distribution and biological characteristics, NPC is distinct from other head and neck cancers [2, 3]. Concurrent chemoradiation therapy (CCRT) is the standard treatment method among local advanced NPC patients [4, 5]. However, there are 6–15% of patients developing distant metastatic lesions before receiving any treatment. Bone, the lung, and the liver are the common metastatic sites [6, 7]. Treatment mostly relies on palliative chemotherapy (PCT) in these patients, with a median overall survival (OS) duration of 12–15 months [8, 9]. However, it was reported that survival rates could exceed 4 years for selected patients with metastatic NPC [10, 11]. Therefore, there is a need to identify the suitable factors for classifying high- or low-risk patients with metastatic NPC. According to National Comprehensive Cancer Network (NCCN) guidelines, chemotherapy combined with locoregional radiotherapy (LRRT) benefits selected patients with distant metastases at limited sites or with low tumor burden [12]. However, which type of patient should receive LRRT remains unclear. If the patients who would benefit from LRRT can be identified, a more intensive treatment strategy could be used to achieve a better clinical outcome.

Several studies have verified that plasma EBV DNA levels measured by real-time quantitative PCR are associated with NPC and can be used as a detection, monitoring, and prognostic prediction marker of non-metastatic NPC [13–17]. Besides, gross tumor volume showed a decreasing trend during treatment. In other types of cancer, tumor response to initial chemotherapy is related to outcome [18–21]. We have also verified the prognostic value of tumor response to neoadjuvant chemotherapy (NACT) among patients with locoregionally advanced NPC [22]. Hence, plasma EBV DNA level and tumor response to chemotherapy might aid the identification of patients who would benefit from LRRT. However, studies regarding plasma EBV DNA level and tumor response in metastatic NPC, especially de novo metastatic NPC are rare. Therefore, we initiated the present study to investigate the prognostic value of these two factors in patients with de novo metastatic NPC and to determine whether radiotherapy benefits these patients based on these two factors.

## Methods

### Patients

Between 2007 and 2016, 502 previously untreated patients with de novo metastatic NPC at our institute were included in the study. The eligibility criteria were: (1) biopsy-proven NPC; (2) received platinum-based PCT; (3) radiologically measurable disease; (4) Karnofsky performance score (KPS) > 60; (5) no secondary pregnancy, lactation, and other malignant disease; (6) normal renal and liver function; (7) available hematological sample results. Routine evaluations were applied on each patient: physical examination, head and neck magnetic resonance imaging (MRI) with contrast, chest radiography/ chest computed tomography (CT), abdominal sonography/ abdominal CT, nasopharyngoscopy and biopsy, bone scan and complete blood count that included differential cell counts, biochemical profile and EBV serology. Positron emission tomography/computed tomography (PET-CT) was also recommended if clinically indicated. The study is approved by Research Ethics Committee of our center.

### Chemotherapy and radiotherapy

All eligible patients underwent one of the following chemotherapy regimens: PF (20–30 mg/m^2^ cisplatin intravenously [IV] on days 1–3 plus 800–1000 mg/m^2^ 5-fluorouracil continuous IV infusion for 24 h on days 1–5), GP (20–30 mg/m^2^ cisplatin IV on days 1–3 plus 800–1000 mg/m^2^ gemcitabine IV on days 1 and 8), TP (75 mg/m^2^ docetaxel IV on day 1 plus 20–25 mg/m^2^ cisplatin IV on days 1–3), TPF (60 mg/m2 docetaxel IV on day 1 plus 20–25 mg/m2 cisplatin IV on days 1–3 plus 500–800 mg/m2 5-fluorouracil continuous IV infusion for 24 h on days 1–5), or other. All regimens were administered every 3 weeks. The median cycle of PCT was five. After PCT, 315 patients were followed by LRRT. Among them, 72 patients received two-dimensional conventional radiotherapy (2D-CRT) and 243 patients received intensity-modulated radiotherapy (IMRT). The median radiation dose to the primary tumor was 70 Gy. The neck tissues received radiotherapy with a median dose of 66 Gy and 54 Gy in metastatic lymph node-positive and lymph node-negative region respectively. All patients were given 5 daily fractions per week and per fraction dose was range from 1.8 to 2.3 Gy. During radiotherapy, 168 patients received cisplatin (60–80 mg/m2)-based concurrent chemotherapy, which was administered every 3 weeks for two radiotherapy cycles [4, 23]. The IMRT plan was designed based on previous studies [24–26].

### Quantification of plasma EBV DNA levels and tumor response assessment

Plasma EBV DNA levels were measured before treatment and after the completion of PCT. Plasma EBV DNA was extracted and subjected to real-time quantitative PCR. We established the post-chemotherapy cut-off value (0 copies/mL) previously [22]. To evaluate the tumor response to therapy, all patients underwent CT, MRI scan or bone scan (for bone metastases) and were classified as having complete response (CR), partial response (PR), stable disease (SD), or progressive disease (PD) based on the Response Evaluation Criteria in Solid Tumors criteria [27]. Patients achieving CR or PR were considered as the satisfactory responders.

### Outcome and follow-up

The primary endpoint of this study was overall survival (OS), which was defined as the time from the date of treatment to the date of death of any cause. After treatment completion, patients were inspected every 3 months in the first 3 years and every 6 months thereafter until death. MRI with contrast of head and neck, CT with contrast of the metastatic sites, Nasopharyngoscopy, chest radiography, abdominal sonography, bone scan, and plasma EBV DNA measurement were routinely performed or upon clinical indication of tumor relapse. PET-CT was considered if necessary.

### Statistical analyses

The statistical analysis was performed using SPSS for Mac version 21.0 (SPSS Inc., Chicago, IL). The relationship between EBV DNA level or tumor response after PCT and the clinical patient characteristics of NPC were evaluated using the χ^2^ test and Fisher’s exact test. Kaplan–Meier survival curves were used to estimate the OS curves; survival rates were compared using the log-rank test. Step-wise multivariate analyses using Cox proportional hazard model were performed with the following variables: post-PCT EBV DNA level, tumor response to PCT, sex, age, T stage, N stage, metastatic site, chemotherapy regimen and LRRT. All analyses were 2-side. The level of significance was set at *P* < 0.05.

## Results

### Clinical characteristics and overall survival

**Fig. 1:**
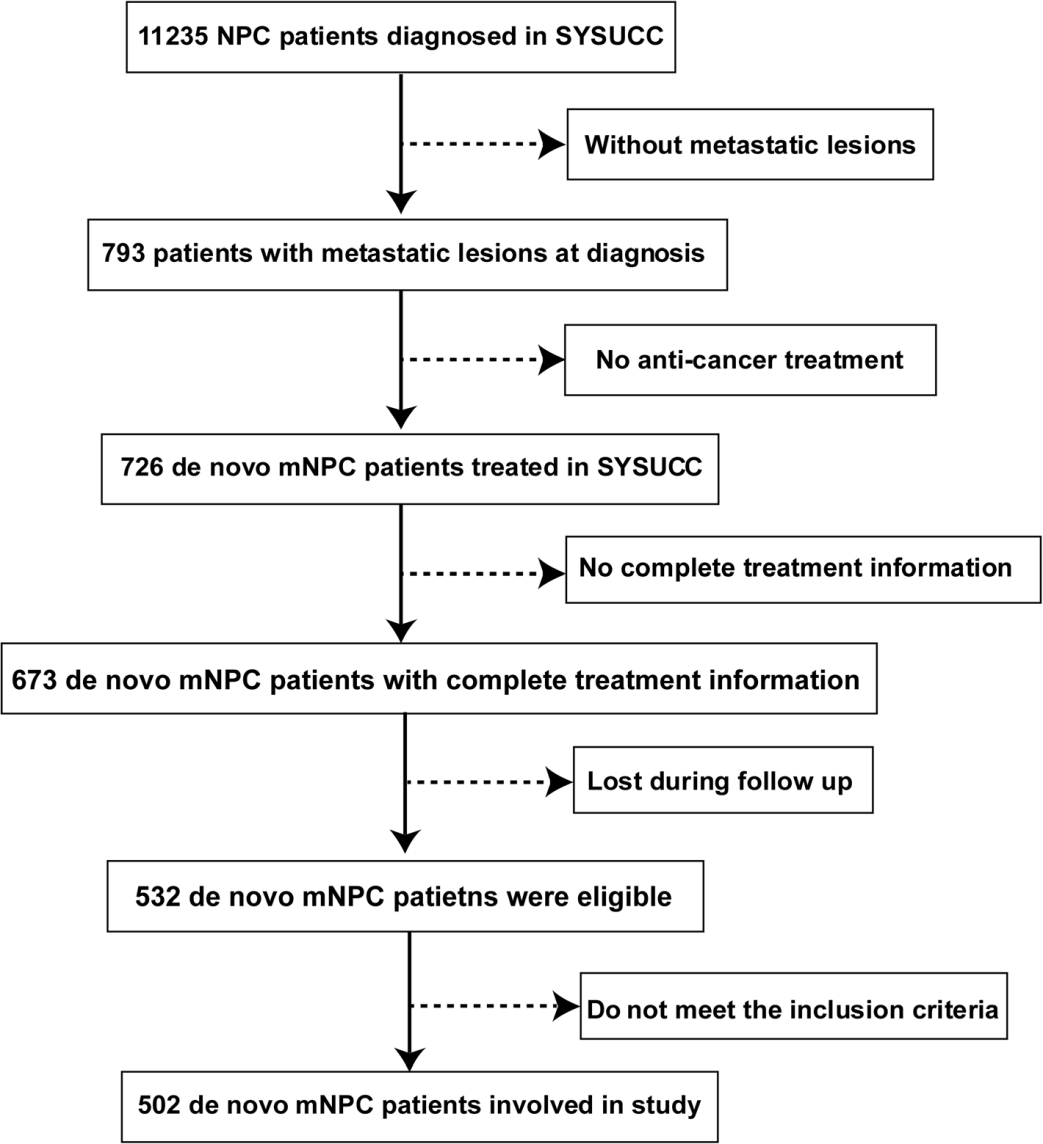
Flow chart of study patient inclusion

Flow chart of patient inclusion was shown in Fig. 1. The median patient age was 47 years (range, 12–74 years); 417 patients (83.1%) were men; 315 patients (62.7%) received LRRT after PCT. In the cohort, 374 patients (74.5%) had one metastatic site, 226 patients (45.0%) had bone-only metastases, 64 patients (12.7%) had lung-only metastases, 49 patients (9.8%) had liver-only metastases, and 35 patients (7.0%) had distant nodal metastases. One hundred and twenty-eight patients (25.5%) had more than one metastatic site. Table 1 lists the characteristics of the 502 patients.

**Table 1:** Clinical characteristics of patients with de novo metastatic NPC (*n* = 502)

	EBV DNA levels after PCT			Tumor response to PCT		
Characteristic	Undetectable	Detectable	*P*-value	CR/PR	SD/PD	*P*-value
Total	249	253		317	185	
Gender						
Male	204 (81.9%)	213 (84.2%)	0.552	260 (82.0%)	157 (84.9%)	0.460
Female	45 (18.1%)	40 (15.8%)		57 (18.0%)	28 (15.1%)	
Age (years)						
≤ 47	133 (53.4%)	128 (50.6%)	0.533	165 (52.1%)	96 (51.9%)	1.000
> 47	116 (46.6%)	125 (49.4%)		152 (47.9%)	89 (48.1%)	
T stage						
T1	10 (4.0%)	11 (4.3%)	1.000	10 (3.2%)	11 (5.9%)	0.467
T2	31 (12.4%)	32 (12.6%)		39 (12.3%)	24 (13.0%)	
T3	123 (49.4%)	125 (49.4%)		161 (50.8%)	87 (47.0%)	
T4	85 (34.1%)	85 (33.6%)		107 (33.8%)	63 (34.1%)	
N stage						
N0	13 (5.2%)	3 (1.2%)	0.002	11 (3.5%)	5 (2.7%)	0.790
N1	53 (21.3%)	36 (14.2%)		55 (17.4%)	34 (18.4%)	
N2	101 (40.6%)	101 (39.9%)		132 (41.6%)	70 (37.8%)	
N3	82 (32.9%)	113 (44.7%)		119 (37.5%)	76 (41.1%)	
Metastatic sites						
Bone	130 (52.2%)	96 (37.9%)	< 0.001	157 (49.5%)	69 (37.3%)	< 0.001
Lung	44 (17.7%)	20 (7.9%)		48 (15.1%)	16 (8.6%)	
Liver	20 (8.0%)	29 (11.5%)		28 (8.8%)	21 (11.4%)	
Distant nodal	23 (9.2%)	12 (4.7%)		27 (8.5%)	8 (4.3%)	
Multiple sites	32 (12.9%)	96 (37.9%)		57 (18.0%)	71 (38.4%)	
PCT regimens						
TPF	77 (30.9%)	53 (20.9%)	0.081	85 (26.8%)	45 (24.3%)	0.639
TP	59 (23.7%)	62 (24.5%)		81 (25.6%)	40 (21.6%)	
PF	63 (25.3%)	68 (26.9%)		78 (24.6%)	53 (28.6%)	
GP	10 (4.0%)	17 (6.7%)		18 (5.7%)	9 (4.9%)	
Others	40 (16.1%)	53 (20.9%)		55 (17.4%)	38 (20.5%)	
LRRT						
Yes	194 (77.9%)	121 (47.8%)	< 0.001	219 (69.1%)	96 (51.9%)	< 0.002
No	55 (22.1%)	132 (52.2%)		98 (30.9%)	89 (48.1%)	

Abbreviations: *EBV* Epstein-Barr virus, *PCT* palliative chemotherapy, *CR* complete response, *PR* partial response, *PD* disease progression, *SD* stable disease, *TPF* cisplatin plus docetaxel plus 5-fluorouracil, *TP* cisplatin plus docetaxel, *PF* cisplatin plus 5-fluorouracil, *GP* cisplatin plus gemcitabine, *LRRT* locoregional radiotherapy

Undetectable/detectable EBV-DNA levels after PCT is based on a cutoff value of 0 copies per milliliter

*P*-value was calculated with the Pearson χ^2^ test

The median follow-up time was 26.3 months (range, 2–126 months). 272 patients died during follow-up. Among them, 269 patients died of tumor progression, 2 patients died of treatment-related toxicities (1 patient because of infection caused by leucopenia and 1 patient because of hepatic failure) and 1 patient died of cardiac disease. EBV DNA levels could be detected in 461 patients before PCT. However, only 253 patients had detectable EBV DNA levels after PCT. Among the entire cohort, two patients had CR, 315 patients had PR, 114 patients had SD, and 71 patients had PD. As shown in Additional file 1: Table S1, the tumor responses were significantly different between patients with post-PCT undetectable plasma EBV DNA levels (CR/PR = 208 of 249 assessable patients) and patients with detectable levels (CR/PR = 109 of 253 assessable patients) (*P* < 0.001).

### Relationship between EBV DNA level, tumor response, and clinical outcome

**Fig. 2:**
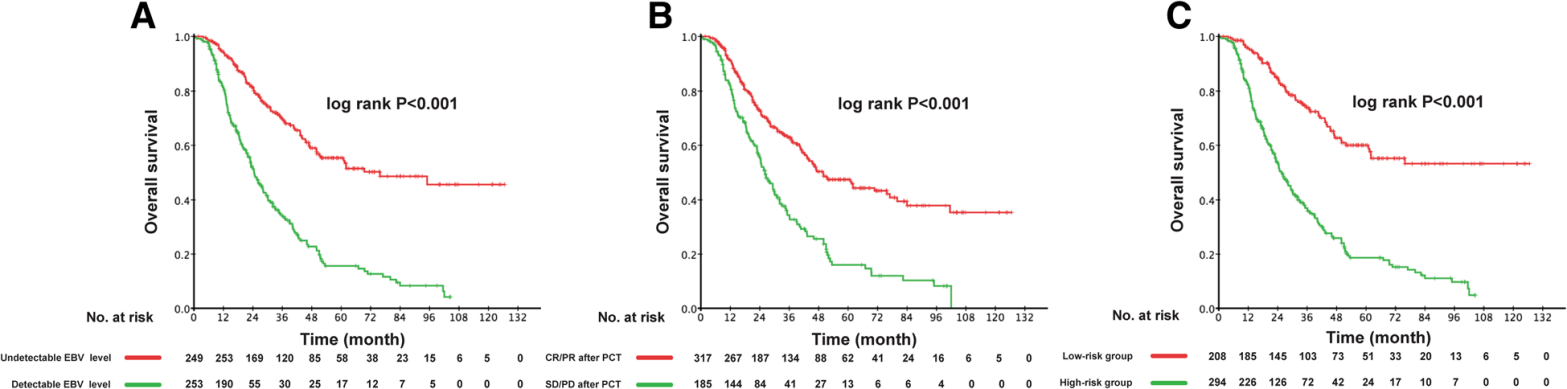
Kaplan–Meier OS curves in 502 patients with de novo metastatic NPC (**a**) Patients grouped according to EBV DNA level after PCT. **b** Patients grouped according to tumor response after PCT. **c** Patients grouped according to risk stratification

The 3-year OS rate in patients with undetectable EBV DNA levels after PCT was significantly higher than that in the patients with detectable EBV DNA levels (69.2, 95% confidence interval [CI] 62.9–75.5% versus 33.8, 95% CI 27.1–40.5%, *P* < 0.001) (Fig. 2a). Unsatisfactory tumor responses predicted worse 3-year OS rate compared with satisfactory responses (62.8, 95% CI 56.9–68.7% versus 33.6, 95% CI 26.0–41.2%, *P* < 0.001) (Fig. 2b). We carried out multivariate analyses that included post-PCT EBV DNA level (undetectable or detectable), post-PCT tumor response (CR/PR or SD/PD), patient age (≤47 years or > 47), sex (male or female), T stage (T_1–2_ or T_3–4_), N stage (N_0–1_ or N_2–3_), chemotherapy regimen (TPF, TP, PF, GP, or other), metastatic site (bone, lung, liver, distant nodal or multiple metastatic sites), and whether PCT was followed by LRRT. Table 2 shows that a significant prognostic value was indicated for both the post-PCT EBV DNA level and tumor response in the multivariate model for OS (EBV DNA level: hazard ratio [HR] 2.13, 95% CI 1.58–2.88, *P* < 0.001; tumor response: HR 1.34, 95% CI 1.02–1.77, *P* = 0.036).

**Table 2:** Multivariate analysis using step-wise method

Characteristic	HR	95% CI	*P*-value
EBV DNA level	2.13	1.58–2.88	< 0.001
Tumor response	1.34	1.02–1.77	0.036
N stage	1.40	1.02–1.91	0.036
Metastatic site			
Lung vs. Bone	0.89	0.58–1.36	0.589
Liver vs. Bone	1.00	0.66–1.51	0.993
Distant nodal vs. Bone	0.98	0.52–1.84	0.956
Multiple vs. Bone	2.39	1.77–3.23	< 0.001
LRRT	0.76	0.59–0.98	0.037

All factors were involved in the analysis and five factors remained

Abbreviations: *EBV* Epstein-Barr virus, *HR* hazard ratio, *CI* confidence interval, *LRRT* locoregional radiotherapy

All potential prognostic factors were involved in the analysis and five factors remained

HRs were calculated for N stage (N2–3 vs. N0–1); LRRT (Yes vs. No); EBV DNA level (Detectable vs. Undetectable) and Tumor response (SD/PD vs. CR/PR)

### Risk stratification

Post-PCT EBV DNA level and tumor response were significantly correlated with prognosis in de novo metastatic NPC. Moreover, the patients with undetectable post-PCT EBV DNA and satisfactory tumor response (CR/PR) had significantly better clinical OS than the patients with either detectable post-PCT EBV DNA or unsatisfactory tumor response (SD/PD); the 3-year OS rates were 73.9 and 36.5% (P < 0.001) respectively (Fig. 2c). Accordingly, we divided the patients into low-risk (undetectable EBV DNA and satisfactory tumor response post-PCT) and high-risk groups (detectable EBV DNA and/or unsatisfactory tumor response post-PCT). Table 3 lists the patient characteristics according to risk group.

**Table 3:** Clinical characteristics of patients with de novo metastatic NPC according to risk after PCT

	Low-risk patients (*n* = 208)			High-risk patients (*n* = 294)		
Characteristic	LRRT	Non-LRRT	*P*-value	LRRT	Non-LRRT	*P*-value
Total	165	43		150	144	
Gender						
Male	134 (81.2%)	34 (79.1%)	0.828	128 (85.3%)	121 (84.0%)	0.871
Female	31 (18.8%)	9 (20.9%)		22 (14.7%)	23 (16.0%)	
Age (years)						
≤ 47	94 (57.0%)	19 (44.2%)	0.169	77 (51.3%)	71 (49.3%)	0.816
> 47	71 (43.0%)	24 (55.8%)		73 (48.7%)	73 (50.7%)	
T stage						
T1	5 (3.0%)	0 (0.0%)	0.495*	6 (4.0%)	10 (6.9%)	0.458
T2	22 (13.3%)	4 (9.3%)		22 (14.7%)	15 (10.4%)	
T3	83 (50.3%)	20 (46.5%)		71 (47.3%)	74 (51.4%)	
T4	55 (33.3%)	19 (44.2%)		51 (34.0%)	45 (31.3%)	
N stage						
N0	9 (5.5%)	0 (0.0%)	0.362	4 (2.7%)	3 (2.1%)	0.452*
N1	33 (20.0%)	8 (18.6%)		29 (19.3%)	19 (13.2%)	
N2	73 (44.2%)	18 (41.9%)		52 (34.7%)	59 (41.0%)	
N3	50 (30.3%)	17 (39.5%)		65 (43.8%)	63 (43.8%)	
Metastatic sites						
Bone	99 (60.0%)	13 (30.2%)	< 0.001*	64 (42.7%)	50 (34.7%)	0.006
Lung	25(%15.2)	13 (30.2%)		15 (10.0%)	11 (7.6%)	
Liver	9 (5.5%)	6 (14.0%)		14 (9.3%)	20 (13.9%)	
Distant nodal	20 (12.1%)	0 (0.0%)		13 (8.7%)	2 (1.4%)	
Multiple sites	12 (7.3%)	11 (25.6%)		44 (29.3%)	61 (42.4%)	
PCT regimens						
TPF	55 (33.3%)	10 (23.3%)	0.021	33 (22.0%)	32 (22.2%)	0.180
TP	46 (27.9%)	7 (16.3%)		39 (26.0%)	29 (20.1%)	
PF	33 (20.0%)	16 (37.2%)		40 (26.7%)	42 (29.2%)	
GP	4 (2.4%)	4 (9.3%)		5 (3.3%)	14 (9.7%)	
Others	27 (16.4%)	6 (14.0%)		33 (22.0%)	27 (18.8%)	

Abbreviations: *LRRT* locoregional radiotherapy, *TPF* cisplatin plus docetaxel plus 5-fluorouracil, *TP* cisplatin plus docetaxel, *PF* cisplatin plus 5-fluorouracil, *GP* cisplatin plus gemcitabine

*P*-value was calculated with the Pearson χ^2^ test or Fisher’s exact test (*)

### The relationship between post-PCT LRRT and outcome in patients according to risk group

**Fig. 3:**
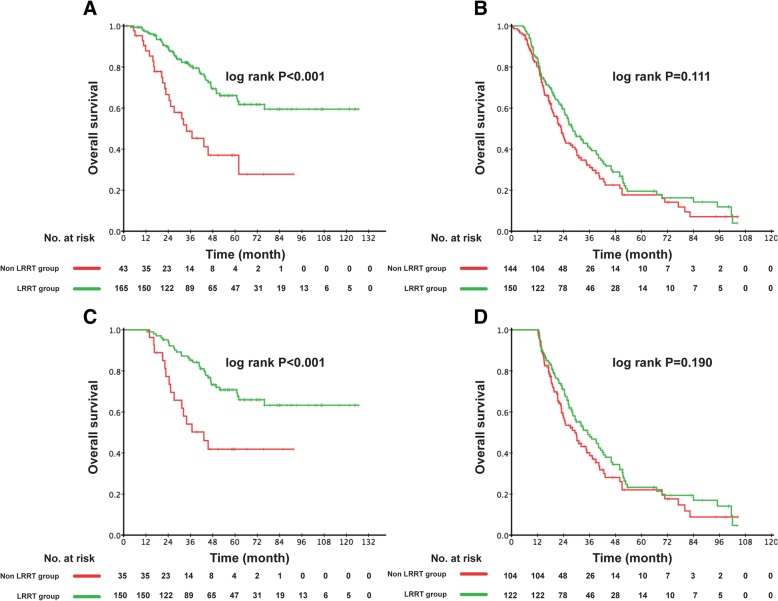
Comparison of OS of patients in the LRRT and non-LRRT group (**a**) Low-risk patients (patients with undetectable EBV DNA level and satisfactory tumor response post-PCT) (**b**) High-risk patients (patients with detectable EBV DNA level or/and unsatisfactory tumor response post-PCT) (**c**) Landmark analyses of overall survival for long-term survivors of ≥1 year in low risk group (**d**) Landmark analyses of overall survival for long-term survivors of ≥1 year in high risk group

In the low-risk group, the 3-year OS rate was 80.4% in LRRT-treated patients and 45.3% in patients not treated with LRRT (*P* < 0.001). However, in the high-risk group, the 3-year OS rate was similar in patients treated or not treated with LRRT (40.2% vs. 31.0%, *P* = 0.111). Figure 3a, b shows the Kaplan–Meier survival curves. Landmark analyses were performed for patients surviving ≥1 year. Similarly, the survival benefit of LRRT for the survivors was only found in low-risk group (Fig. 3c, d). We carried out two separate multivariate analyses of the low- and high-risk patients. Table 4 shows that in the low-risk group, a strong prognostic value was indicated for LRRT for OS (HR 0.35, 95% CI 0.21–0.58, *P* < 0.001). However, LRRT did not show significant survival benefits for the high-risk group.

**Table 4:** Multivariate analysis of OS in low- and high-risk patients after PCT using step-wise method

	Low-risk patients			High-risk patients		
Characteristic	HR	95% CI	*P*-value	HR	95% CI	*P*-value
Age (years)			NS	1.27	0.96–1.68	0.089
N stage	1.85	0.99–3.45	0.055	1.38	0.97–1.96	0.074
Metastatic site						
Lung vs. Bone			NS	0.83	0.48–1.42	0.493
Liver vs. Bone			NS	1.03	0.65–1.62	0.911
Distant nodal vs. Bone			NS	0.90	0.43–1.87	0.774
Multiple vs. Bone			NS	2.94	2.10–4.11	< 0.001
LRRT	0.35	0.21–0.58	< 0.001			NS

Abbreviations: *NS* non-significant, *HR* hazard ratio, *CI* confidence interval, *LRRT* locoregional radiotherapy

All potential prognostic factors were involved in the analysis. Two factors remained in low-risk patients and three factors remained in high-risk patients

HRs were calculated for Age (years) (>47 vs. ≤47); N stage (N2–3 vs. N0–1); LRRT (Yes vs. No)

## Discussion

Distant metastasis has been a leading cause of death in patients with NPC, and there is no effective treatment for such patients [28, 29]. The management of metastatic NPC remains a crucial clinical challenge. The development of modern treatment modalities (e.g., IMRT, tomotherapy, targeted therapy, biotherapy) makes individualized treatment increasingly important, as these treatments are expensive and toxic. PCT is usually recommended for improving long-term survival. However, the benefits of LRRT in different types of patients with de novo metastatic NPC have not been clearly evaluated. This lack of understanding has hindered individualized treatment of metastatic NPC. There is a need to integrate biomarkers and diagnostic imaging assays to determine the risk of poor outcome in individual patients and the type of patient who would benefit from subsequent radiotherapy. Thus, we divided patients with de novo metastatic NPC into high- and low-risk groups based on EBV DNA level and tumor response to PCT, and found that post-PCT LRRT could significantly reduce the risk of death in low-risk patients.

Many reports have proven the value of LRRT in patients with metastatic NPC. Among patients with distant metastases of NPC at diagnosis, Chen et al. proved that patients who received LRRT plus systemic chemotherapy had higher survival rates compared with patients who received systemic chemotherapy alone [30]. According to Khot et al., patients with NPC with bone-only oligometastatic lesions could benefit from LRRT [31]. Moreover, the NCCN guidelines state that patients with metastatic NPC with limited sites or with low tumor burden would benefit from LRRT [12]. Under the background, Zou et al. divided patients with de novo metastatic NPC into three subgroups according survival outcome: M1a, no liver involvement and oligometastases; M1b, no liver involvement and multiple metastases; and M1c, liver involvement regardless of the number of distant metastases. They reported that LRRT combined with chemotherapy benefited patients identified as M1a/M1b but did not result in better clinical outcome for patients classified as M1c [32]. In our study, 315 patients were followed by LRRT after PCT and 187 patients received PCT only. The median cycle of PCT was five (three weeks a cycle). Between chemotherapy and RT initiation, the median of time intervals were 21 days (range: 10–38 days). There is potential for immortal time bias. Only patients that survive long enough can actually receive radiation and patients that die or progress before they can get LRRT cannot possibly receive LRRT. The observations of consistent benefits with RT on landmark analyses suggest that this form of bias could not affect our results.

Plasma EBV DNA level and tumor response correlate with the tumor burden and prognosis [13–15]. In the present study, patients with undetectable plasma EBV DNA levels after PCT had significantly better clinical outcome than patients with detectable EBV DNA levels. Similarly, satisfactory tumor response predicted better OS compared with unsatisfactory response. The results are accordance with our previous study of patients with locoregionally advanced NPC [22]. For patients with metastatic or recurrent NPC, An et al. have suggested that undetectable post-treatment plasma EBV DNA indicates better tumor response [33]. Moreover, it was inspiring to observe that patients in our study with both undetectable plasma EBV DNA and satisfactory tumor response post-PCT had a 3-year OS rate of 73.9%, which is significantly higher than that in patients with detectable EBV DNA and/or unsatisfactory tumor response post-PCT (36.5%). Accordingly, considering these two factors, we divided the patients into low-risk (undetectable EBV DNA and satisfactory tumor response post-PCT) and high-risk groups (detectable EBV DNA and/or unsatisfactory tumor response post-PCT). We explored the efficacy of additional post-PCT LRRT in the patients according to risk group. Interestingly, in the low-risk group, patients who received post-PCT LRRT had significantly better OS compared to the patients who did not. This might be because the low-risk patients were sensitive to PCT and that the distant lesions were under better control. Local control may be important to long-term survival in this kind of patients. Therefore, it is more important to control the locoregional disease by post-PCT LRRT. However, for high-risk patients, the distant lesions were not under control and the post-PCT LRRT might have been insufficient for controlling the distant lesions. Intensive treatment such as the administration of target agent or the addition of immunotherapy might be necessary for such patients to control the distant lesions [34, 35]. Therefore, we did not observe a significant survival benefit of post-PCT LRRT in these patients.

There are several limitations to this study. First, the study involved only 502 patients, and the number of patients between subgroups differed which is an unavoidable bias because of the small sample size. Second, the data were from one center; the results should be validated by a multi-centric clinical study. The third limitation is that the median follow-up duration was 26.3 months. A longer follow-up period is necessary to evaluate the long-term outcomes of these patients and to validate our results.

## Conclusion

In conclusion, EBV DNA level and tumor response after PCT are closely related with the clinical outcome of de novo metastatic NPC. Patients with undetectable EBV DNA and satisfactory tumor response after PCT could benefit from the addition post-PCT LRRT. This finding would identify patients at different risk of treatment failure and guide individualized therapy. Further investigation is necessary to confirm our findings.

## Additional file

Additional file 1: **Table S1.** Relationship between EBV DNA levels after PCT and tumor response to PCT. (DOCX 49 kb)

## Abbreviations

2D-CRT: Two-dimensional conventional radiotherapy
CI: Confidence interval
CR: Complete response
CT: Computed tomography
EBV: Epstein–Barr virus
HR: Hazard ratio
IMRT: Intensity-modulated radiotherapy
KPS: Karnofsky performance score
LRRT: Locoregional radiotherapy
MRI: Magnetic resonance imaging
NCCN: National Comprehensive Cancer Network
NPC: Nasopharyngeal carcinoma
OS: Overall survival
PCT: Palliative chemotherapy
PD: Progressive disease
PET-CT: Positron emission tomography/computed tomography
PR: Partial response
SD: Stable disease
