# Supplementary material for: Identifying optimal candidates for local treatment of the primary tumor among patients with de novo metastatic nasopharyngeal carcinoma: a retrospective cohort study based on Epstein–Barr virus DNA level and tumor response to palliative chemotherapy
Source: BMC Cancer. 2019 Jan 21;19:92. doi: 10.1186/s12885-019-5281-5 (PMC6341516; doi:10.1186/s12885-019-5281-5)
Supplement: Supplementary file 1 — Table S1. Relationship between EBV DNA levels after PCT and tumor response to PCT. (DOCX 49 kb) [file 12885_2019_5281_MOESM1_ESM.docx]

Table S1 Relationship between EBV DNA levels after PCT and tumor response to PCT

|  | **Tumor response to PCT** | | |
| --- | --- | --- | --- |
| **EBV-DNA level** | CR/PR | SD/PD | *P*-value |
| **Undetectable** | 208 (65.6%) | 41 (22.2%) | <0.001 |
| **Detectable** | 109 (34.4%) | 144 (77.8%) |  |

EBV = Epstein-Barr virus; PCT = palliative chemotherapy

Undetectable/detectable EBV-DNA levels after PCT is based on a cutoff value of 0 copies per milliliter.

*P*-value was calculated with the Pearson χ^2^ test
